# Development and validation of a UPLC-MS/MS method with volumetric absorptive microsampling to quantitate cyclophosphamide and 4-hydroxycyclophosphamide

**DOI:** 10.3389/fphar.2022.928721

**Published:** 2022-08-11

**Authors:** Yahdiana Harahap, Steven Steven, Herman Suryadi

**Affiliations:** ^1^ Bioavailability/Bioequivalence Laboratory, Faculty of Pharmacy, Universitas Indonesia, Depok, Indonesia; ^2^ Faculty of Military Pharmacy, Republic of Indonesia Defense University, Sentul, Indonesia

**Keywords:** cyclophosphamide, 4-hydroxycyclophosphamide, 4-hydroxycyclophosphamide-d4, LC-MS/MS, volumetric absorptive microsampling

## Abstract

Cyclophosphamide (CP) is an anti-cancer alkylating prodrug, metabolized by CYP450 into its active metabolite 4-hydroxycyclophosphamide (4-OHCP). Its therapeutic effectiveness is determined by the 4-OHCP concentration. Several analytical methods in plasma and dried blood spots have been developed to analyze cyclophosphamide and 4-OHCP; however, there are many disadvantages. The objective of this study was to develop and validate the ultraperformance liquid chromatography–tandem mass spectrometry (UPLC–MS/MS) method by volumetric absorptive microsampling (VAMS) and 4-hydroxycyclophosphamide-d4 (4-OHCP-d4) as an internal standard. VAMS requires small sample volumes, and it is not affected by the hematocrit values; therefore, it is an efficient sampling method. The samples were derivatized with 5 μL semicarbazide hydrochloride (SCZ) and 25 μL of the resulting 4-OHCP-SCZ; 4-OHCP-d4-SCZ derivatives were absorbed by VAMS and extracted by protein precipitation. The optimum conditions were obtained using the Waters Acquity^®^ UPLC BEH C18 (2.1 × 100 mm; 1.7 μm) column; flow rate 0.15 ml/min; mobile phase 0.01% formic acid and methanol; gradient elution mode for 6 min by positive electrospray ionization; and multiple reaction monitoring of m/z 260.7 > 140.0 for CP, 333.7 > 221.0 for 4-OHCP-SCZ, and 337.7 > 225.1 for 4-OHCP-d4-SCZ. The method met the validation requirements set by the FDA. The cyclophosphamide LLOQ value was 5 ng/mL, and the calibration curve range was 5—60,000 ng/ml. Furthermore, the 4-OHCP LLOQ value was 2.5 ng/ml, and the calibration curve range was 2.5—1,000 ng/ml.

## 1 Introduction

Cyclophosphamide (CP) is a prodrug which needs to be metabolized to become the active metabolite, 4-hydroxycyclophosphamide (4-OHCP). The metabolic process starts with an oxidation reaction by the CYP450 enzyme. Then, 4-OHCP undergoes tautomerization to become aldophosphamide until it reaches the equilibrium stage; then, it is metabolized spontaneously by non-enzymatic reactions to phosphoramide mustard and acrolein. Phosphoramide mustard provides a therapeutic effect by forming covalent bonds with the N7 site on the guanine base, forming cross-links, and ultimately breaking the DNA strands (Ahlmann and Hempel, 2016).

The therapeutic doses of CP were categorized into low dose (1–3 mg/kg), moderate dose (15–40 mg/kg), and high dose (>120 mg/kg) groups. The possibility of side effects of acute toxicity to occur in patients who are administered high doses of CP is higher. In addition, long-term therapy (>6 months) has also been shown to cause side effects of chronic toxicity. The toxicities that can be caused by CP are cardiotoxicity, hematotoxicity, and gonadotoxicity (Emadi, Jones, & Brodsky, 2009). The onset of toxic effects in patients receiving CP is influenced by the narrow therapeutic window and large interindividual pharmacokinetic variability (Paci et al., 2019). Therefore, it is necessary to do therapeutic drug monitoring (TDM) by determining the level of drugs and their metabolites in the blood. This needs to be performed to optimize drug efficacy and minimize drug side effects (Ehab and Desoky, 2018).

In previous research studies, CP and 4-OHCP analysis methods were developed in plasma samples and dried blood spots (DBS) by ultra-high-performance liquid chromatography–tandem mass spectrometry or ultraperformance liquid chromatography–tandem mass spectrometry (UPLC–MS/MS) (Hall et al., 2018; Harahap et al., 2020). Currently, UPLC–MS/MS has been more often used in bioanalysis because it can provide more sensitive, selective, and high throughput results than other instruments (Harmita et al., 2019). In the analysis using plasma samples, the lower limit of quantification (LLOQ) was as follows, 34.3 ng/ml for CP and 3.42 ng/ml for 4-OHCP (Hall et al., 2018). Meanwhile, research with DBS obtained the LLOQ as low as 50 ng/ml for CP and 10 ng/ml for 4-OHCP (Harahap et al., 2020). Plasma sampling using the venipuncture technique requires a large volume of blood (3–5 ml). Meanwhile, the amount of samples obtained by using the DBS method may vary because it is influenced by the hematocrit effect which can cause differences in the area of the spots on the DBS paper (Daousani et al., 2019). Therefore, a new method of analysis and bio sampling that is more effective, efficient, and sensitive was needed to analyze CP and 4-OHCP.

Over the last few years, the new microsampling method volumetric absorptive microsampling (VAMS) has been developed to overcome the shortcomings of the previous sampling methods. As low as 10 μL of blood can be drawn within 2–4 s using the VAMS method with a maximum possible volume variation of 5% (Denni & Spooner, 2014). VAMS is performed using the finger-prick technique so that the procedure is simple and does not cause patient discomfort. In addition, the VAMS method is not affected by the hematocrit effect, so the sample volume does not vary (Protti et al., 2018). Despite its advantages, the CP and 4-OHCP analysis methods using VAMS have never been developed.

In this study, the development and validation of an UPLC–MS/MS method using VAMS are reported to quantitate CP and 4-OHCP. The method can be applied to measure CP and 4-OHCP in patients’ samples who received CP for efficient TDM.

## 2 Materials and methods

### 2.1 Chemicals and reagents

Cyclophosphamide (100%) (Sigma Aldrich, St. Louis, MO, United States), 4-hydroxycyclophosphamide kit (98%) (Toronto Research Chemical, North York, ON, Canada), and 4-hydroxycyclophosphamide-d4 kit (95%) as internal standards (IS) (Toronto Research Chemical, North York, ON, Canada), and semicarbazide hydrochloride (Sigma Aldrich, St. Louis, MO, United States), acetonitrile (99.9%) HPLC grade, methanol (99.9%) HPLC grade, formic acid (98—100%), and ethyl acetate (99.5%) were purchased from Merck (Darmstadt, Germany); whole blood was purchased from the Indonesian Red Cross (Jakarta, Indonesia), ultrapure water from Sartorius Water Filter System, and VAMS (Neoteryx^®^, Torrance, CA, United States).

### 2.2 UPLC–MS/MS

The UPLC–MS/MS system consisted of a Waters Acquity^®^ UPLC H-Class Quatenary Solvent Manager, a Waters Acquity^®^ UPLC Sample Manager FTN, a Waters XEVO TQD triple quadrupole mass spectrometer equipped with a Zspray™ source by positive electrospray ionization. The analytes were detected with multiple reaction monitoring with m/z 260.7 > 140.0 for CP, m/z 333.7 > 221.0 for 4-OHCP-SCZ, and m/z 337.7 > 225.1 4-OHCP-d4-SCZ IS. The system was controlled by MassLynx version 4.1. The detailed mass spectrometric conditions are available in the Supplementary Material.

The analytes were separated on a Waters Acquity^®^ UPLC C18 BEH (2.1 × 100 mm; 1.7 μm) column using a mobile phase consisting of 0.01% formic acid in water and methanol with gradient elution. The flow rate was 0.15 ml/min, column temperature 50°C, injection volume 10 μL, and run time 6 min. The detailed mobile phase optimization and gradient programming data are available in the Supplementary Material.

### 2.3 Preparation of stock and working solutions

Here, 2 mg/ml stock solution of CP was made in ultrapure water. A measure of 5 mg/ml 4-OHCP stock solution was prepared by dissolving 5.0 mg of sodium thiosulfate with 1.0 ml of ultrapure water and adding the solution to a vial that contained 5.0 mg of 4-OHCP. A 2.5 mg/ml 4-OHCP-d4 stock solution was prepared by dissolving 2.5 mg of sodium thiosulfate with 1.0 ml of ultrapure water and adding the solution to a vial that contained 2.5 mg of 4-OHCP. Working solutions should be prepared by diluting the stock solutions with ultrapure water to yield the following concentrations, 100 μg/ml, 1,000 μg/ml, and 1,500 μg/ml for CP; 10 μg/ml and 1,000 μg/ml for 4-OHCP; and 1 μg/ml for 4-OHCP-d4.

### 2.4 Preparation of calibrators and quality control samples

Calibrators should be prepared by diluting working solutions with whole blood until the concentration obtained 5—60,000 ng/ml for CP and 2.5—1,000 ng/ml for 4-OHCP. Quality control (QC) samples should be prepared by diluting working solutions with whole blood until the concentration reached 15 ng/ml (QCL), 24,000 ng/ml (QCM), and 45,000 (QCH) for CP, while for 4-OHCP 7.5 ng/ml (QCL), 400 ng/ml (QCM), and 750 ng/ml (QCH). The SCZ standard was dissolved with 10 ml of 50 mmol potassium phosphate (pH 7.4) buffer until 2 M concentration was acquired.

### 2.5 Sample preparation with VAMS

Five μL of the 2 M SCZ solution was prepared using VAMS by immersing the tip in the derivatization solution. The VAMS tip was dried for 2 h, and then 25 µL of blood containing CP and 4-OHCP was also added into VAMS. The tips were then dried at room temperature for 2 h. The tip was removed from the handle and placed on the microtube. A total of 20 μL of the internal standard solution with a concentration of 1 μg/mL and 1,000 μL of methanol were added to the microtube. The mixture was vortex for 30 s, sonicated for 10 min, and centrifuged at 10,000 rpm for 10 min. A total of 900 μL of the supernatant was taken and evaporated by blowing N2 gas for 15 min at 60°C. The residue was reconstituted with 100 μL of 0.01% formic acid—methanol (50:50, v/v), vortexed for 30 s, sonicated for 2 min, and centrifuged at 10,000 rpm for 10 min, and 10 μL was injected to the UPLC–MS/MS.

### 2.6 Method validation

Validation of this analysis method was based on the Food and Drug Administration (FDA) Bioanalytical Method Validation (Food and Drug Administration U.S. Department of Health Services, 2018).

### 2.7 Lower limit of quantification

The lowest non-zero calibrators on the calibration curve which showed at least five times the response of the zero calibrators should be considered the LLOQ. The accuracy should not exceed ±20% of nominal concentration and %CV should not exceed 20% from ≥5 replicates in at least three runs.

### 2.8 Calibration curve

A blank, a zero sample, and seven calibrator levels (including the LLOQ) covering the quantitation range should be prepared in the same matrix as the study samples and should be used in every run. The accuracy of the non-zero calibrators should be ±15% of the nominal concentration, except LLOQ which should be ±20% in each validated run. At least 75% or seven of the non-zero calibrator levels should meet the criteria.

### 2.9 Selectivity

Blank and zero calibrators in the LLOQ concentration of the appropriate biological matrix from at least six individual sources should be prepared and should be free of interference at the analyte and internal standard (IS) retention times. The blank sample response should not exceed ±20% of LLOQ and ±5% of IS.

### 2.10 Accuracy and precision

Accuracy and precision tests should be performed at least three times for both within-run and between-run by using ≥5 replicates of LLOQ, QCL, QCM, and QCH concentrations for each run. The acceptance criterion for accuracy is ±15% of the nominal concentration, except LLOQ which should be ±20%. The % CV for the precision criterion should not exceed 15%, except for LLOQ which should not exceed 20%.

### 2.11 Recovery

The area results from the extracted samples at LLOQ, QCL, QCM, and QCH concentrations were compared with the area from extracts of the blanks spiked with the analyte after the extraction at those four concentrations. The recovery should be consistent and reproducible.

### 2.12 Carryover and matrix effect

Carryover was carried out by first injecting the ULOQ concentration samples, followed by blank sample injection. Carryover should not exceed 20% of LLOQ. As for the matrix effect, the area response of the analytes at low concentration (maximum three times of LLOQ), high concentration (maximum ULOQ), and IS spiked in six different individual sample sources was compared with the area diluted in the solvents. % CV of the samples should not exceed 15%, and the normalized matrix factor should be in the 0.8–1.2 range.

### 2.13 Stability

The sample stability test was carried out with several conditions, such as auto-sampler, bench-top, stock solution, and long-term stability test, each performed at least thrice at QCL and QCH concentrations. The CP stock solution was tested in 2–8°C, while the 4-OHCP and 4-OHCP-d4 stock solutions in −80°C, both for the long-term stability test. The short-term stock solution stability test was carried out at 25°C (room temperature). A long-term stability test was carried out at −80°C and 25°C, each for 30 days. A bench-top stability test was conducted at 25°C for 24 h. An autosampler stability test was conducted for 24 h.

## 3 Results and discussion

The objective of this study was to develop and validate a UPLC–MS/MS method for the quantification of CP, 4-OHCP using VAMS, and 4-OHCP-d4 as an IS. The method was validated according to FDA Bioanalytical Method Validation guidelines (Food and Drug Administration U.S. Department of Health Services, 2018). The detailed method optimization parameters are available in the Supplementary Material.

### 3.1 Method validation

#### 3.1.1 Calibration curve

The range of the calibration curve for CP and 4-OHCP was designed according to the literature data (Hasanah, 2020). The calibrators used for CP were 5, 10, 100, 1,000, 10,000, 30,000, and 60,000 ng/ml. Meanwhile, the calibrators for 4-OHCP were 2.50, 5, 10, 50, 100, 500, and 1,000 ng/ml. The results of the CP and 4-OHCP calibration curves for the with-in run and between run met the requirements. With this linear calibration curve, the proportional results of the actual analyte concentration within the specific range can be achieved and is considered reliable. The results are presented in Supplementary Table S18. The range of the calibration curves was wider than those presented in the literature (Harahap et al., 2016), which provides easier quantification of CP and 4-OHCP and may eliminate the need for sample dilution.

#### 3.1.2 LLOQ and selectivity

**FIGURE 1:**
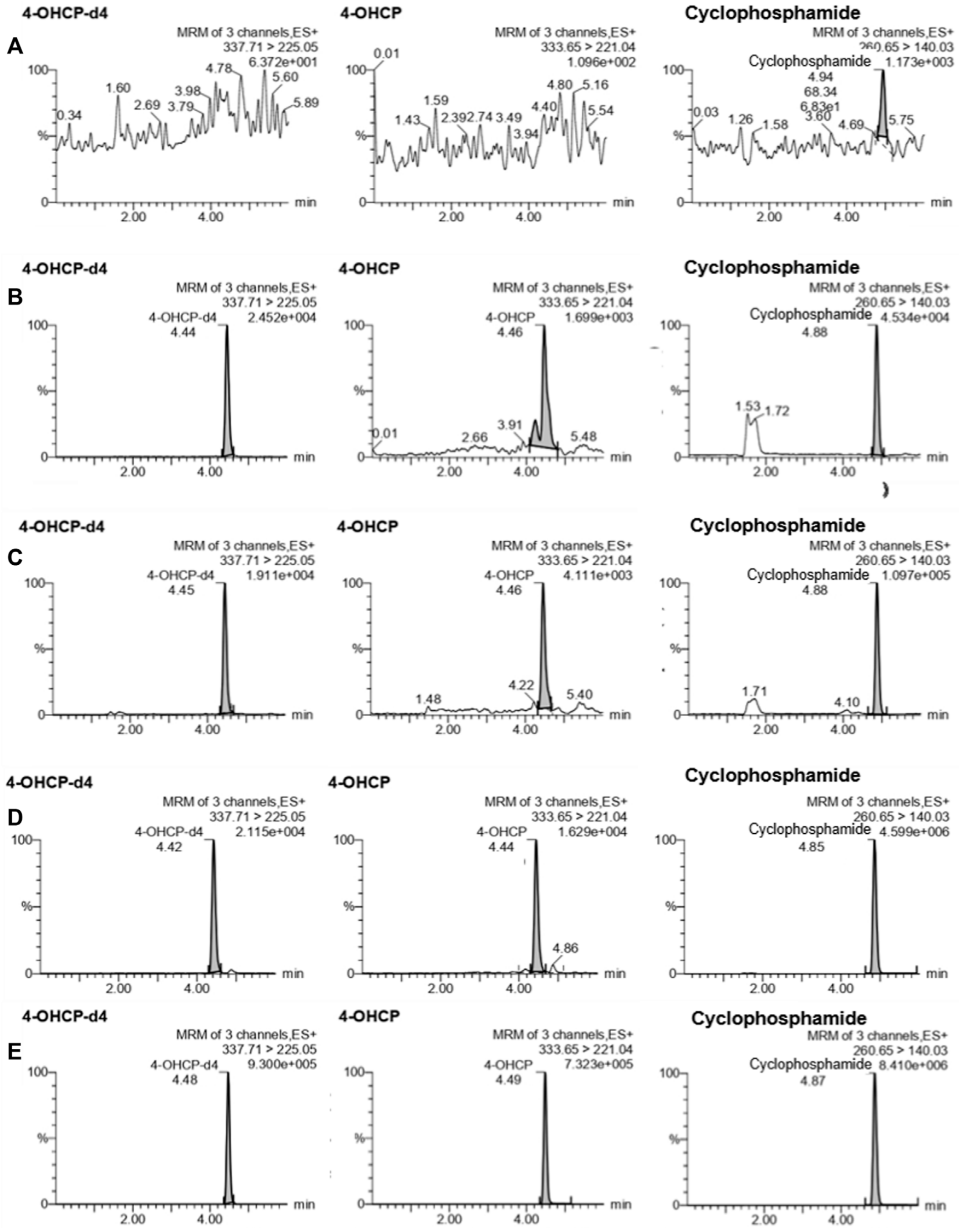
Representative UPLC–MS/MS chromatograms of cyclophosphamide, 4-hydroxycyclophosphamide (4-OHCP), and 4-hydroxycyclophosphamide-d4 (4-OHCP-d4) in **(A)** blank VAMS; VAMS spiked with analyte at **(B)** LLOQ; **(C)** QCL; **(D)** QCM; and **(E)** QCH.

The results of the LLOQ determination are presented in Table 1. The LLOQ was 5 ng/ml for CP and 2.5 ng/ml for 4-OHCP, and they met the acceptance criteria for accuracy and precision. These results represent an improved LLOQ compared to studies where DBS (Hasanah, 2020) or plasma (Harahap et al., 2016) were used. The LLOQ obtained with the present method is lower which may be because of the variance of the samples obtained with VAMS is low (Denni & Spooner, 2014) and the sample collection is not affected by the hematocrit values (Protti et al., 2018). The results for selectivity met the acceptance criteria. The percentage interference ranged between 1.87% and 2.55% for CP, 6.99% and 14.2% for 4-OCHP, and 0.830% and 1.80% for 4-OHCP-d4. In comparison with studies using DBS, the variances were higher (Hasanah, 2020); therefore, the current method using VAMS is considered as more selective than those using DBS. Representative UPLC–MS/MS chromatograms of blank VAMS and VAMS spiked with the analytes at LLOQ are shown in Figures 1A,B.

**TABLE 1:** LLOQ accuracy and precision.

Analyte	LLOQ concentration (ng/mL)	Measured concentration (ng/mL)	Accuracy (%diff)	Precision (%CV)
Cyclophosphamide	5	4.62	−7.61	10.7
		5.58	11.5	
		4.57	−8.70	
		5.17	3.43	
		4.30	−14.0	
4-OHCP	2.5	2.46	−1.55	10.3
		2.24	−10.2	
		2.84	13.6	
		2.51	0.28	
		2.22	−11.4	

#### 3.1.3 Accuracy and precision

The results for accuracy and precision are presented in Table 2. Intra-day accuracy ranged between −13.4% and 13.9% for CP and −13.8% and 15% for 4-OHCP; precision ranged between 4.19% and 9.91% for CP, 6.16% and 9.24% for 4-OHCP; inter-day accuracy ranged between −14.9% and 14.2% for CP, −14.7% and 19.1% for 4-OHCP 9 (LLOQ); and precision ranged between 6.14% and 8.85% for CP, 6.94% and 9.12% for 4-OHCP. The results for accuracy and precision met the acceptance criteria, and the method is considered accurate and precise. Representative UPLC–MS/MS chromatograms of the samples of a blank VAMS and samples spiked with the analytes at LLOQ, QCL, QCM, and QCH are shown in Figures 1A–E.

**TABLE 2:** Intra- and inter-day accuracies and precision.

Analyte	Concentration (ng/ml)	Intra-day		Inter-day	
		Mean accuracy (%diff)	Precision (%CV)	Mean accuracy (%diff)	Precision (%CV)
Cyclophosphamide	5	−10.1%–12.9%	9.74	−13.9%–12.9%	8.85
	15	−13.4%–14.0%	9.91	−14.7%–14.0%	8.58
	24.000	2.75%–13.9%	4.19	−14.9%–14.2%	8.27
	45.000	−8.29%–9.96%	7.01	−9.82%–12.3%	6.14
4-OHCP	2.5	−10.1%–9.48%	8.34	−14.7%–19.1%	9.12
	7.5	−6.73%–14.5%	7.78	−13.5%–14.9%	8.77
	400	−3.05%–10.1%	6.16	−11.8%–12.6%	6.94
	750	−13.8%–10.8%	9.24	−13.8%–10.8%	7.29

#### 3.1.4 Recovery and carryover

The percentage recovery ranged between 0.56% and 1.79% for CP, 0.44% and 1.69% for 4-OHCP. The results met the acceptance criteria, and it is concluded that the sample preparation was efficient and reproducible. The recovery values were higher than those obtained from plasma (Rahma, 2014), but not much different from those using DBS (Hasanah, 2020), however, the current method using VAMs yielded a smaller %CV, therefore, the precision is considered better. The carry-over ranged between 10.7% and 14.0% for CP, 13.7% and 19.1% for 4-OHCP, and 2.47% and 4.52% for 4-OHCP-d4. The results met the acceptance criteria, and it is concluded that the effect of a high concentration injection will not interfere with the subsequent injection.

#### 3.1.5 Matrix effect

The results met the acceptance of % CV not more than 15% and the normalized IS matrix factor values were between 0.8 and 1.2, which indicated that there was no significant effect from the matrix and the results obtained can be considered reliable. The results were compared to methods using DBS (Hasanah, 2020) and plasma (Rahma, 2014) and found that with the current method using VAMS, the normalized matrix factor value had an average closer to 1 and a lower % CV; therefore, it is concluded that the matrix effect obtained with DBS and plasma were greater than those obtained with VAMS.

#### 3.1.6 Stability

The stability results are presented in Supplementary Tables S19, 20. The short-term (24 h), long-term (30 days), stock solution, bench-top VAMS, long-term VAMS, and autosampler stability met the acceptance criteria for CP, 4-OHCP, and 4-OHCP-d4. The long-term VAMS stability test was performed at -80°C and at room temperature (25°C). The results showed that the VAMS samples can be stored at room temperature making sample storage and distribution more convenient. The storage of the VAMS samples at -80°C was more stable that is consistent with the storing conditions for 4-OHCP and 4-OHCP-d4 (Santa Cruz Biotechnology, 2016; National Center for Biotechnology Information, 2020), while CP and 4-OHCP were stable in DBS at -80°C for 7 days only (Hasanah, 2020). Therefore, the VAMS method is considered more stable than the DBS method.

#### 3.1.7 System suitability test

The system suitability test was carried out under optimum analytical conditions in order to ensure that the instruments and methods obtained perform properly during the analysis. The test should be carried out right before the analysis every day in 5 replicates by using the mixture of CP, 4-OHCP, and 4-OHCP-d4 with a concentration of 1 μg/ml. The acceptance criteria were the % CV of retention time and the peak area of each analyte to be below 6% (Briscoe et al., 2007). The results obtained met the requirements, thus the system is considered to perform well. The results for the system suitability test are shown in Supplementary Table S8.

## 4 Conclusion

A UPLC–MS/MS method to quantify CP and 4-OHCP was developed and validated successfully. The method showed better sensitivity and selectivity compared to previously developed methods; therefore, it is concluded that the current method offers an improved analytical tool to analyze CP and 4-OHCP using VAMS. Also, it can be implemented in surveillance regimes of CP therapy under TDM.

## Data Availability

The original contributions presented in the study are included in the article/Supplementary Material; further inquiries can be directed to the corresponding author.
