# Supplementary material for: Development and validation of a UPLC-MS/MS method with volumetric absorptive microsampling to quantitate cyclophosphamide and 4-hydroxycyclophosphamide
Source: Front Pharmacol. 2022 Aug 11;13:928721. doi: 10.3389/fphar.2022.928721 (PMC9403605; doi:10.3389/fphar.2022.928721)
Supplement: Supplementary file 1 [file Table1.DOCX]

# SUPPLEMENTARY MATERIAL

## Method development and optimization

### Optimization of mass condition

Optimum mass spectrometry conditions were achieved with Multiple Reaction Monitoring (MRM) and positive ionization method (ESI +). MRM mode was chosen because this mode has high sensitivity and selectivity, also it can select a specific m/z value and can be used to detect more than one analyte (You *et al*., 2013). Meanwhile, the ESI + method was chosen because the analyte and standard are basic compounds. The analyte and internal standard can be protonated (addition ion H^+^) to produce parent ions which are then fragmented into daughter ions (Banerjee & Mazumdar, 2011). Parameters of the mass spectrometry that was optimized are capillary voltage which was 3.50 kV; gas desolvation temperature was 350°C; gas desolvation flow rate was 400 L/hour; cone voltage was 34 V, 16 V, and 20 V respectively for cyclophosphamide, 4-hydrocyclophosphamide-semicarbazone (4-OHCP-SCZ), and 4-hydrocyclophosphamide-d_4_-semicarbazone (4-OHCP-d_4_-SCZ); and collision voltage was 20 V, 12 V, and 8 V respectively for cyclophosphamide, 4-OHCP-SCZ, and 4-OHCP-d_4_-SCZ. The parent ion and daughter ion m/z values were 260.65>140.03 for cyclophosphamide, 333.65>221.04 for 4-OHCP-SCZ, and 337.71>225.05 for 4-OHCP-d_4_-SCZ. The results for optimization of mass spectrometry condition are shown in **Table S1**. The fragmentation spectrums are shown in **Figure S1**.

### Optimization of mobile phase combination

Mobile phase combination optimization was carried out by comparing 4 variations, which are (a) 0.1% acetic acid solution - acetonitrile, (b) 0.1% acetic acid solution - methanol, (c) 0.01% formic acid solution - acetonitrile, and (d) 0.01% formic acid solution - methanol. This test was performed under isocratic conditions with a flow rate of 0.2 mL/minute, the composition of the mobile phase was 50:50 (v/v), and the column temperature was 50°C. The results for optimization of mobile phase combination are shown in **Table S2**. Based on the area obtained, the mobile phase combination (b) produced the largest area followed by the mobile phase combination (d). The addition of 0.1% acetic acid in the mobile phase as the aqueous phase produces a larger area when compared to 0.01% formic acid. However, 0.01% formic acid is more commonly chosen because it has a smaller concentration of iron and other metal contaminants compared to 0.1% acetic acid. Iron and other metal contaminants can affect the analysis results and damage the column (Waters, 2008). The mobile phase combination using acetonitrile has a faster retention time but a smaller area, while the mobile phase combination using methanol has a longer retention time but a larger area. The mobile phase combination using acetonitrile has a lower viscosity value than the mobile phase combination using methanol. Viscosity has an influence on column pressure. The smaller the viscosity, the lower the column pressure, and vice versa k (Harmita *et al*., 2019). The lower column pressure can increase efficiency so that the retention time is faster (Majors, 2007). The mobile phase combinations (b) and (d) have their own advantages so that the researcher decided to continue these two combinations to the next optimization stage.

### Optimization of mobile phase composition

Mobile phase composition optimization was carried out by comparing 4 variations for mobile phase combinations (b) and (d), which are 60:40, 50:50, 40:60, and 30:70 (v/v). The results for optimization of mobile phase composition are shown in **Table S3**. As for the mobile phase combination (b), the 50:50 (v/v) composition was chosen because it could produce the largest area. On the other hand, for mobile phase composition (d), it was found that the 50:50 (v/v) composition could produce the largest 4-OHCP and 4-OHCP-d4 areas, while the largest cyclophosphamide area was produced by the composition 40:60 (v/v). Although the resulting area for cyclophosphamide at a composition of 40:60 (v/v) was larger, a researcher still chose the 50:50 (v/v) composition because of the significant differences in the 4-OHCP and 4-OHCP-d4 areas when compared to the composition 40:60 (v/v). Based on the retention time, the greater concentration of acid composition, the longer the retention time would be. Between the results gathered from mobile phase combination and composition optimization, the results were the same, so both of the mobile phase combinations, (b) and (d), were continued to the next optimization stage.

### Optimization of mobile phase gradient elution

Mobile phase gradient elution optimization was carried out by comparing 3 variations, which are shown in **Table S4**. The results for optimization of mobile phase gradient elution are shown in **Table S5**. Profile 3 of the combination (b) showed the largest area. However, the resulting area is not too different between profiles 1, 2, and 3. Profile 1 has the shortest and profile 3 has the longest retention time, but it was still considered efficient because the run time was under 6 minutes. Therefore, profile 3 was selected. The result from gradient condition analysis was greater than the isocratic condition. Thus, in the combination of mobile phases (b) a gradient elution analysis was chosen.

Then, in the combination of the mobile phase (d), the largest cyclophosphamide area was obtained in profile 2 and the smallest was in profile 3. Meanwhile, the area 4-OHCP and 4-OHCP-d4 in profile 2 and profile 3 did not differ significantly. Based on these data, the researcher chose profile 2 in the combination of the mobile phase (d). The result obtained from the gradient condition was significantly greater than from the isocratic gradient, thus in the combination (d), gradient elution mode was chosen.

The elution profiles selected for each mobile phase combination (b) and (d) were compared, analysis with gradient elution in combination (d) yields a larger and significantly different area of ​​cyclophosphamide. However, the resulting areas for 4-OHCP and 4-OHCP-d4 did not differ significantly. In addition, combination (d) was more efficient than combination (b) because it has a shorter retention time. Besides, the combination of the mobile phase (b) was using 0.1% acetic acid which has a greater potential to damage the column compared to the combination of the mobile phase (d) which used 0.01% formic acid (Waters, 2007). Therefore, elution profile 2 was chosen with a combination of mobile phase (d), namely formic acid 0.01% - methanol.

### Optimization of flow rate

Flow rate optimization was carried out under optimum results obtained from the previous optimization, namely gradient elution profile 2 with a mobile phase of 0.01% formic acid - methanol. There are three variations of the flow rate, which were 0.15 mL/minute, 0.20 mL/minute, and 0.25 mL/minute. The results for optimization of flow rate are shown in **Table S6**.

Based on the area of ​​the analyte and the internal standard, the smaller the flow rate, the larger the area produced, and vice versa. The flow rate of 0.15 mL/minute produced the largest area. Despite the fact that the smaller the flow rate, the longer the analyte elutes, 0.15 mL/minute was still chosen because it produces a larger area and the retention time is still relatively short, which was under 5 minutes. In addition, flow rate selection is also based on the consideration that a slower flow rate might decrease the column pressure which can affect the column lifespan because the higher the column pressure, the faster the column will be damaged (Harmita *et al.*, 2019)

### Optimization of column temperature

The column temperature was optimized after obtaining the optimum conditions from the previous optimization results. There are three variations in column temperature, which were 30°C, 40°C, and 50°C. The results for optimization of column temperature are shown in **Table S7**. Based on the data obtained, the higher the column temperature, the larger area produced. However, changes in column temperature did not affect the retention time of the analyte. In addition, an increase in column temperature can reduce column pressure due to a decrease in the viscosity of the mobile phase (Majors, 2007). Therefore, the column temperature of 50°C was chosen as the optimum column temperature.

## Optimization of sample preparation

### Optimization of extraction method

There are three variations which are protein precipitation, liquid-liquid extraction (LLE), and a combination of protein precipitation and LLE. The difference was in the extraction solution used. The protein precipitation used methanol, the liquid-liquid extraction used ethyl acetate - acetonitrile (50:50, v/v), while the combination method used the mixture of the two methods. The results for optimization of extraction method are shown in **Table S9**. The result showed that the protein precipitation produced the largest response and more efficient than the other two methods because the extracting solution used does not need to be prepared and is easier to obtain. In the liquid-liquid extraction method, the partition between nonpolar and polar liquids was difficult to distinguish so that high accuracy was required. Meanwhile, the combination method required a long preparation time and the area produced was the smallest among all. Therefore, the optimum extraction method was protein precipitation.

### Optimization of derivatization method

The derivatization reaction of aldophosphamide/4-OHCP with semicarbazide hydrochloride is shown in **Figure S2**. The results for optimization of derivatization method are shown in **Table S10**. The data showed that the third derivatization method produced the largest area, while the first derivatization method produced the smallest area. This was because in the first derivatization method, the sample was dried on the VAMS tip for 2 hours and after that, the derivatization solution was added. Meanwhile, the 4-OHCP compound is very unstable so that during the drying time of 4-OHCP it can undergo hydrolysis to become acrolein and phosphoramide mustard (Hall *et al*., 2018). Furthermore, the second derivatization method also produced a small area. This could be because after the VAMS was prepared with derivatized solutions, the tips were immediately used to absorb blood. In this process, the derivatized solution that has not fully dried could mix with the blood so that the volume between the blood and the derivatization solution cannot be ascertained. Therefore, the third method, which was done by absorbing the derivatization solution on the VAMS and drying it first for 2 hours before being used for sampling, was chosen.

### Optimization of derivatization solution volume

The derivatization solution volume, 2 M semicarbazide HCl was optimized between three variations, which are 5 μL, 10 μL, and 15 μL. The results for optimization of derivatization solution volume are shown in **Table S11**. The more the derivatization solution volume, the less the sample volume could be absorbed. This is because VAMS has its own capacity and can only absorb a certain amount of sample volume. The volume of VAMS being used was 30 μL. Based on the test results, the derivatization solution volume of 5 μL produced the largest area compared to the others. The result also showed that the less the derivatization solution, the larger area obtained. This might due to the increased amount of blood and analytes being absorbed. Therefore, the derivatization volume of 5 μL was chosen.

### Optimization of VAMS drying time

The drying time was optimized with three variations which are 1 hour, 2 hours and 3 hours. The results for optimization of VAMS drying time are shown in **Table S12**. Based on the result, 2 hours of drying time produced the largest area, while 1 hour was the smallest. This was because the sample was not completely dry with just an hour of drying time, so the sample was susceptible to lose during the tip removal process and produced the smallest area. The area result with 3 hours of drying time was smaller and not significantly different from 2 hours. Therefore, it could be concluded that the optimum drying time of the sample with VAMS was 2 hours. Drying time above 2 hours might cause analyte degradation because of longer exposure to the air. Moreover, the longer the drying time, the more difficult it is to extract the analyte because the analyte is fully absorbed into VAMS.

### Optimization of extraction solution

The optimum extraction method that resulted from the optimization, was the protein precipitation method. Therefore, during this optimization, the extraction solution for the protein precipitation method (methanol and acetonitrile) was used. Optimization was carried out in five variations, namely methanol, methanol-acetonitrile (2:1), methanol-acetonitrile (1:1), methanol-acetonitrile (1:2), and acetonitrile. The results for optimization of extraction solution are shown in **Table S13**. The largest area was obtained with methanol as the extraction solution because the analytes could be fully dissolved in a methanol-only solution. The higher the acetonitrile concentration, the smaller the area produced (Food and Drug Administration, 2016; Toronto Research Chemicals, 2019). In addition, the methanol has a smaller boiling point, which is 64.6°C, compared to acetonitrile, which is 81.65°C, thus the time required for the evaporation process with methanol is shorter and the sample preparation process is more efficient (Joshi & Adhikari, 2019). Therefore, the composition of the extraction solution chosen is methanol.

### Optimization of extraction solution volume

The volume of the extraction solution was optimized with three variations, which are 500 μL, 750 μL, and 1,000 μL. The results for optimization of extraction solution volume are shown in **Table S14**. The higher volume of the extraction solution resulted in the greater amount of analyte extracted thus the larger the area produced. Therefore, 1000 μL of extraction solution was chosen as the optimum volume.

### Optimization of vortex time

The vortex time was optimized with three variations which are 15, 30, and 45 seconds. The results for optimization of vortex time are shown in **Table S15**. The vortex aimed to maximize the analyte absorption into the extraction solution. The largest area was produced with 30 seconds of the vortex.

### Optimization of sonication time

The sonication time was optimized with three variations, which are 5, 10, and 15 minutes. Sonication aimed to maximize the analyte absorption by utilizing ultrasonic frequencies. The results for optimization of vortex time are shown in **Table S16**. The sonication for 5 minutes produced the smallest area because too short of sonication time caused nonoptimal analyte extraction. Then, due to the insignificant difference in results between sonication for 10 minutes and 15 minutes, a shorter time was chosen, which is 10 minutes.

### Optimization of centrifugation time

Centrifugation in this sample preparation aimed to precipitate proteins and other impurities in order to obtain a clear supernatant containing analytes. The centrifugation speed used was 10,000 rpm and the variations were 5, 10, and 15 minutes. The results for optimization of vortex time are shown in **Table S17**. Centrifugation for 5 minutes produced the smallest area because there were contaminants interfering with the analysis results due to the nonoptimal deposition process. When compared with centrifugation for 10 and 15 minutes, the resulting area was larger, but not significantly different. Therefore, the optimal centrifugation time chosen was 10 minutes.

# References

1. Food and Drug Administration. Bioanalytical Method Validation. 2018.

2. Hall OM, Peer CJ, Fitzhugh CD, Figg WD. A Sensitive and Rapid Ultra High-Performance Liquid Chromatography with Tandem Mass Spectrometric Assay for the Simultaneous Quantitation of Cyclophosphamide and the 4-Hydroxycyclophosphamide Metabolite in Human Plasma. *J Chromatogr B*. 2018;1086(March):56-62. doi:10.1016/j.jchromb.2018.04.016

3. Harmita AAK, Harahap Y, Supandi. *Liquid Chromatography-Tandem Mass Spectrometry (LC-MS/MS)*.; 2019.

4. Majors RE. Column Pressure Considerations in Analytical HPLC. *LCGC North Carolina*. 2007;25(11):1074-1092.

5. Joshi DR, Adhikari N. An Overview on Common Organic Solvents and Their Toxicity. *J Pharm Res Int*. 2019:1-18. doi:10.9734/jpri/2019/v28i330203

6. Toronto Research Chemicals. (2019). 4-Hydroxycyclophosphamide. Retrieved from https://www.trc-canada.com/product-detail/?H926302

7. Waters. Controlling Contamination in UltraPerformance LC/MS and HPLC/MS Systems. *Waters Corp*. 2008. https://www.waters.com/webassets/cms/support/docs/715001307d_cntrl_cntm.pdf.

8. You J, Willcox MD, Madigan MC, et al. *Tear Fluid Protein Biomarkers*. Vol 62. 1st ed. Elsevier Inc.; 2013. doi:10.1016/B978-0-12-800096-0.00004-4

**Table S1** Mass spectrometry conditions optimization data

| Compound | Ion Fragment (*m/z*) | Capillary Tube Pressure (kV) | Desolvation Gas Temperature | Desolvation Gas Flow Rate | *Cone* (V) | *Collision* (V) |
| --- | --- | --- | --- | --- | --- | --- |
| CP | 260.65 > 140.03 | 3,0 | 450 | 500 | 34 | 20 |
| 4-OHCP-SCZ | 333.65 > 221.04 |  |  |  | 16 | 12 |
| 4-OHCP-d_4_-SCZ | 337.71 > 225.05 |  |  |  | 20 | 8 |

**Table S2** Mobile phase combination optimization data

| **Mobile Phase** | **Area (µV/s)** | | | **Retention Time (minute)** | | |
| --- | --- | --- | --- | --- | --- | --- |
|  | CP | 4-OHCP | 4-OHCP-d_4_ | CP | 4-OHCP | 4-OHCP-d_4_ |
| **Acetic Acid** 0**.**1% - A**cetonitrile** | 147,630.469 | 15,099.433 | 27,338.121 | 1.75 | 1.36 | 1.38 |
| **Acetic Acid** 0**.**1% - Met**h**anol | 234,417.906 | 37,335.879 | 73,367.953 | 3.01 | 2.15 | 2.13 |
| **Formic Acid** 0**.**01% - A**cetonitrile** | 163,605.266 | 11,958.160 | 21,487.895 | 1.61 | 1.33 | 1.33 |
| **Formic Acid** 0**.**01% - Met**h**anol | 219.,304.094 | 26,727.287 | 54,355.156 | 2.74 | 1.93 | 1.93 |

**Table S3** Mobile phase composition optimization data

1. Acetic Acid 0.1% - Methanol

| **Composition Acetic Acid** 0**.**1% - Met**h**anol | **Area (µV/s)** | | | **Retention Time (minute)** | | |
| --- | --- | --- | --- | --- | --- | --- |
|  | CP | 4-OHCP | 4-OHCP-d_4_ | CP | 4-OHCP | 4-OHCP-d_4_ |
| 60:40 (v/v) | 180,166.875 | 31,504.629 | 66,083.000 | 4.56 | 2.68 | 2.66 |
| 50:50 (v/v) | 231,847.656 | 37,099.156 | 71,801.984 | 3.00 | 2.13 | 2.12 |
| 40:60 (v/v) | 168,157.188 | 22,791.984 | 45,349.512 | 2.22 | 1.80 | 1.79 |
| 30:70 (v/v) | 159,262.938 | 14,805.773 | 26,728.908 | 1.82 | 1.61 | 1.61 |

1. Formic Acid 0.01% - Methanol

| **Composition Formic Acid 0.01% - Methanol** | **Area (µV/s)** | | | **Retention Time (minute)** | | |
| --- | --- | --- | --- | --- | --- | --- |
|  | CP | 4-OHCP | 4-OHCP-d_4_ | CP | 4-OHCP | 4-OHCP-d_4_ |
| 60:40 (v/v) | 150,859.906 | 20,095.354 | 41,471.641 | 3.94 | 2.39 | 2.37 |
| 50:50 (v/v) | 220,415.047 | 26,179.338 | 53,342.184 | 2.74 | 1.93 | 1.92 |
| 40:60 (v/v) | 237,418.422 | 20,324.486 | 38,575.461 | 2.07 | 1.67 | 1.67 |
| 30:70 (v/v) | 197,284.922 | 13,600.865 | 24,400.051 | 1.66 | 1.46 | 1.46 |

**Table S4** Gradient elution profile

| Min to- | Profile 1 | | Profile 2 | | Profile 3 | |
| --- | --- | --- | --- | --- | --- | --- |
|  | Mobile phase A (%) | Mobile phase B (%) | Mobile phase A (%) | Mobile phase B (%) | Mobile phase A (%) | Mobile phase B (%) |
| 0 | 70 | 30 | 80 | 20 | 90 | 10 |
| 1 | 10 | 90 | 10 | 90 | 10 | 90 |
| 2 | 10 | 90 | 10 | 90 | 10 | 90 |
| 2.5 | 70 | 30 | 80 | 20 | 90 | 10 |
| 6 | 70 | 30 | 80 | 20 | 90 | 10 |

**Table S5** Mobile phase gradient elution optimization data

| **Acetic Acid 0.1% - Methanol** | | | | | | |
| --- | --- | --- | --- | --- | --- | --- |
| **Gradient Elution Profile** | **Area (µV/s)** | | | **Retention Time (minute)** | | |
|  | CP | 4-OHCP | 4-OHCP-d_4_ | CP | 4-OHCP | 4-OHCP-d_4_ |
| 1 | 248,127.219 | 48,504.965 | 85,977.367 | 3.40 | 2.88 | 2.86 |
| 2 | 245,592.156 | 55,352.719 | 101,020.414 | 3.75 | 3.40 | 3.39 |
| 3 | 247,703.781 | 62,371.316 | 110,269.391 | 3.91 | 3.63 | 3.62 |
| **Formic Acid 0.01% - Methanol** | | | | | | |
| 1 | 333,630.563 | 47,044.488 | 76,139.461 | 3.35 | 2.79 | 2.76 |
| 2 | 341,221.813 | 56,963.285 | 94,434.820 | 3.70 | 3.36 | 3.35 |
| 3 | 294,556.469 | 63,748.531 | 103,810.711 | 4.07 | 3.69 | 3.68 |

**Table S6** Flow rate optimization data

| **Flow Rate (mL/min)** | **Area (µV/s)** | | | **Retention Time (minute)** | | |
| --- | --- | --- | --- | --- | --- | --- |
|  | CP | 4-OHCP | 4-OHCP-d_4_ | CP | 4-OHCP | 4-OHCP-d_4_ |
| 0**.**15 | 442,656.813 | 80,258.719 | 122,128.797 | 4.89 | 4.47 | 4.46 |
| 0**.**20 | 348,005.719 | 57,535.621 | 93,894.445 | 3.71 | 3.37 | 3.36 |
| 0**.**25 | 262,318.344 | 54,154.750 | 85,045.164 | 3.14 | 2.89 | 2.88 |

**Table S7** Column temperature optimization data

| **Column Temperature (°C)** | **Area (µV/s)** | | | **Retention Time (minute)** | | |
| --- | --- | --- | --- | --- | --- | --- |
|  | CP | 4-OHCP | 4-OHCP-d_4_ | CP | 4-OHCP | 4-OHCP-d_4_ |
| 30 | 336,432.438 | 70,150.617 | 113,044.750 | 4.86 | 4.45 | 4.44 |
| 40 | 343,431.906 | 70,456.898 | 112,619.797 | 4.91 | 4.50 | 4.49 |
| 50 | 441,735.625 | 78,112.836 | 118,414.766 | 4.89 | 4.47 | 4.46 |

**Table S8** Data of system suitability test

| **Data** | **Area (µV/s)** | | | **Retention Time (minute)** | | |
| --- | --- | --- | --- | --- | --- | --- |
|  | CP | 4-OHCP | 4-OHCP-d_4_ | CP | 4-OHCP | 4-OHCP-d_4_ |
| 1 | 425,651.000 | 73,295.914 | 86,609.336 | 4.90 | 4.47 | 4.48 |
| 2 | 423,915.969 | 72,472.758 | 86,176.328 | 4.91 | 4.48 | 4.48 |
| 3 | 423,051.188 | 70,488.070 | 83,917.516 | 4.91 | 4.48 | 4.48 |
| 4 | 434,283.969 | 72,651.188 | 84,986.203 | 4.91 | 4.49 | 4.48 |
| 5 | 431,689.469 | 71,932.797 | 84,773.734 | 4.91 | 4.48 | 4.48 |
| **Average** | 427,718.319 | 72,168.145 | 85,292.623 | 4.91 | 4.48 | 4.48 |
| **SD** | 4,984.77 | 1,057.83 | 1,091.87 | 0.004 | 0.007 | 0.000 |
| **%CV** | 1.17 | 1.47 | 1.28 | 0.09 | 0.16 | 0.00 |

**Table S9** Extraction method optimization data

| **Extraction Method** | **Area (µV/s)** | | | **Retention Time (minute)** | | |
| --- | --- | --- | --- | --- | --- | --- |
|  | CP | 4-OHCP | 4-OHCP-d_4_ | CP | 4-OHCP | 4-OHCP-d_4_ |
| **Liquid – Liquid Extraction (LLE)** | 13,513.611 | 886.839 | 920.659 | 4.94 | 4.50 | 4.49 |
| **Protein Precipitation (PP)** | 28,738.804 | 921.416 | 1,261.889 | 4.94 | 4.51 | 4.50 |
| **PP and LLE** | 12,793.745 | 752.527 | 906.615 | 4.94 | 4.51 | 4.50 |

**Table S10** Derivatization method optimization data

| **Derivatization Method** | **Area (µV/s)** | | | **Retention Time (minute)** | | |
| --- | --- | --- | --- | --- | --- | --- |
|  | CP | 4-OHCP | 4-OHCP-d_4_ | CP | 4-OHCP | 4-OHCP-d_4_ |
| **Method 1** | 28,460.248 | 942.088 | 1,238.657 | 4.91 | 4.48 | 4.47 |
| **Method 2** | 28,093.523 | 1,056.922 | 1,181.519 | 4.91 | 4.48 | 4.47 |
| **Method 3** | 29,714.323 | 1,131.857 | 1,252.167 | 4.90 | 4.48 | 4.47 |

**Table S11** Derivatization solution volume optimization data

| **Derivatization Solution Volume (µL)** | **Area (µV/s)** | | | **Retention Time (minute)** | | |
| --- | --- | --- | --- | --- | --- | --- |
|  | CP | 4-OHCP | 4-OHCP-d_4_ | CP | 4-OHCP | 4-OHCP-d_4_ |
| 5 | 35,513.664 | 1,965.278 | 1,552.849 | 4.89 | 4.46 | 4.45 |
| 10 | 32,451.531 | 1,176.527 | 1,501.181 | 4.91 | 4.48 | 4.47 |
| 15 | 30,860.021 | 915.101 | 1,509.151 | 4.91 | 4.48 | 4.47 |

**Table S12** VAMS drying time optimization data

| **VAMS Drying Time (hour)** | **Area (µV/s)** | | | **Retention Time (minute)** | | |
| --- | --- | --- | --- | --- | --- | --- |
|  | CP | 4-OHCP | 4-OHCP-d_4_ | CP | 4-OHCP | 4-OHCP-d_4_ |
| 1 | 30,592.924 | 1,274.620 | 1,592.172 | 4.89 | 4.46 | 4.46 |
| 2 | 35,138.271 | 1,919.694 | 1,550.696 | 4.89 | 4.46 | 4.45 |
| 3 | 34,398.782 | 1,730.379 | 1,431.833 | 4.89 | 4.46 | 4.45 |

**Table S13** Extraction solution optimization data

| **Extraction Solution** | **Area (µV/s)** | | | **Retention Time (minute)** | | |
| --- | --- | --- | --- | --- | --- | --- |
|  | CP | 4-OHCP | 4-OHCP-d_4_ | CP | 4-OHCP | 4-OHCP-d_4_ |
| Met**h**anol | 36,926.767 | 1,821.293 | 1,558.713 | 4.89 | 4.46 | 4.45 |
| Met**h**anol – A**cetonitrile** (2:1) | 36,058.183 | 1,645.001 | 1,529.691 | 4.89 | 4.46 | 4.45 |
| Met**h**anol – A**cetonitrile** (1:1) | 35,605.599 | 1,615.288 | 1,553.840 | 4.88 | 4.45 | 4.44 |
| Met**h**anol – A**cetonitrile** (1:2) | 35,556.030 | 1,580.833 | 1,497.611 | 4.88 | 4.45 | 4.44 |
| A**cetonitrile** | 35,635.221 | 1,302.087 | 1,440.912 | 4.89 | 4.46 | 4.45 |

**Table S14** Extraction solution volume optimization data

| **Extraction Solution Volume (µL)** | **Area (µV/s)** | | | **Retention Time (minute)** | | |
| --- | --- | --- | --- | --- | --- | --- |
|  | CP | 4-OHCP | 4-OHCP-d_4_ | CP | 4-OHCP | 4-OHCP-d_4_ |
| 500 | 37,186.023 | 1,508.986 | 1,523.014 | 4.88 | 4.45 | 4.44 |
| 750 | 36,846.466 | 1,687.637 | 1,591.891 | 4.89 | 4.46 | 4.45 |
| 1**,**000 | 41,468.470 | 1,982.638 | 1,642.495 | 4.88 | 4.46 | 4.45 |

**Table S15** Vortex time optimization data

| **Vortex Time (second)** | **Area (µV/s)** | | | **Retention Time (minute)** | | |
| --- | --- | --- | --- | --- | --- | --- |
|  | CP | 4-OHCP | 4-OHCP-d_4_ | CP | 4-OHCP | 4-OHCP-d_4_ |
| 15 | 38,210.924 | 1,751.216 | 1,491.197 | 4.87 | 4.45 | 4.44 |
| 30 | 42,150.405 | 2,041.268 | 1,662.152 | 4.88 | 4.46 | 4.45 |
| 45 | 40,375.687 | 1,905.995 | 1,608.056 | 4.88 | 4.45 | 4.44 |

**Table S16** Sonication time optimization data

| **Sonication Time (minute)** | **Area (µV/s)** | | | **Retention Time (minute)** | | |
| --- | --- | --- | --- | --- | --- | --- |
|  | CP | 4-OHCP | 4-OHCP-d_4_ | CP | 4-OHCP | 4-OHCP-d_4_ |
| 5 | 39,912.421 | 1,802.586 | 1,575.044 | 4.86 | 4.44 | 4.43 |
| 10 | 41,444.843 | 2,130.331 | 1,621.842 | 4.88 | 4.45 | 4.44 |
| 15 | 42,182.516 | 2,093.278 | 1,597.299 | 4.87 | 4.44 | 4.43 |

**Table S17** Centrifugation optimization data

| **Centrifugation Time (minute)** | **Area (µV/s)** | | | **Retention Time (minute)** | | |
| --- | --- | --- | --- | --- | --- | --- |
|  | CP | 4-OHCP | 4-OHCP-d_4_ | CP | 4-OHCP | 4-OHCP-d_4_ |
| 5 | 33,020.018 | 1,182.094 | 1,475.211 | 4.86 | 4.44 | 4.43 |
| 10 | 41,804.325 | 2,230.037 | 1,761.535 | 4.88 | 4.45 | 4.44 |
| 15 | 43,072.658 | 2,180.010 | 1,687.320 | 4.87 | 4.44 | 4.43 |

**Table S18** Data of inter-day calibration curve

| **Analyte** | **Replicate inter-day** | **R** | **Slope (b)** | **Intercept (a)** |
| --- | --- | --- | --- | --- |
| Cyclophosphamide | 1 | 0.99989 | 0.01236 | 4.46572 |
|  | 2 | 0.99991 | 0.01235 | 4.43728 |
|  | 3 | 0.99991 | 0.01235 | 4.35842 |
|  | **Mean** | **0.99990** | **0.01235** | **4.42047** |
| 4-OHCP | 1 | 0.99651 | 0.00165 | 0.08029 |
|  | 2 | 0.99706 | 0.00166 | 0.09037 |
|  | 3 | 0.99696 | 0.00165 | 0.08170 |
|  | **Mean** | **0.99684** | **0.00165** | **0.08411** |

**Table S19** The stability test result of Cyclophosphamide and 4-OHCP stock solution

| **Stability Test** | **Average %diff of the Concentration** | | |
| --- | --- | --- | --- |
|  | **Cyclophosphamide** | **4-OHCP** | **4-OHCP-d_4_** |
| Short term (24 h, 25^o^C) | Hour 0: 0.00%  Hour 24: -1.41% | Hour 0: 0.00%  Hour 24: -2.06% | Hour 0: 0.00%  Hour 24: -2.34% |
| Long term (30 days, -4^o^C for Cyclophosphamide, -80^o^C for 4-OHCP and 4-OHCP-d_4_) | Hour 0: 0.00%  Hour 24: -1.60% | Hour 0: 0.00%  Hour 24: -2.65% | Hour 0: 0.00%  Hour 24: -1.52% |

**Table S20** The stability test result of whole blood spiked with Cyclophosphamide and 4-OHCP on VAMS

| **Stability Test** | **Average %diff of the Concentration** | | | |
| --- | --- | --- | --- | --- |
|  | **Cyclophosphamide** | | **4-OHCP** | |
|  | **QCL (15 ng/mL)** | **QCH (45,000 ng/mL)** | **QCL (7.50 ng/mL)** | **QCH (750 ng/mL)** |
| Autosampler (24 h) | Hour 0: 2.60%  Hour 24: 1.63% | Hour 0: 2.38%  Hour 24: -4.61% | Hour 0: 1.38%  Hour 24: -2.62% | Hour 0: 1.46%  Hour 24: -2.60% |
| Bench-top (24 h, 25^o^C) | Hour 0: 2.60%  Hour 24: -4.17% | Hour 0: 2.38%  Hour 24: -0.24% | Hour 0: 1.35%  Hour 24: 1.46% | Hour 0: 1.46%  Hour 24: -2.45% |
| Long term (30 days, deep freezer -80^o^C) | Day 0: 2.60%  Day 30: -6.92% | Day 0: 2.38%  Day 30: -4.33% | Day 0: 1.35%  Day 30: -6.18% | Day 0: 1.46%  Day 30: -5.51% |
| Long term (30 days, 25^o^C) | Day 0: 2.60%  Day 30: -8.39% | Day 0: 2.38%  Day 30: -7.58% | Day 0: 1.35%  Day 30: -12.38% | Day 0: 1.46%  Day 30: -12.76% |

**
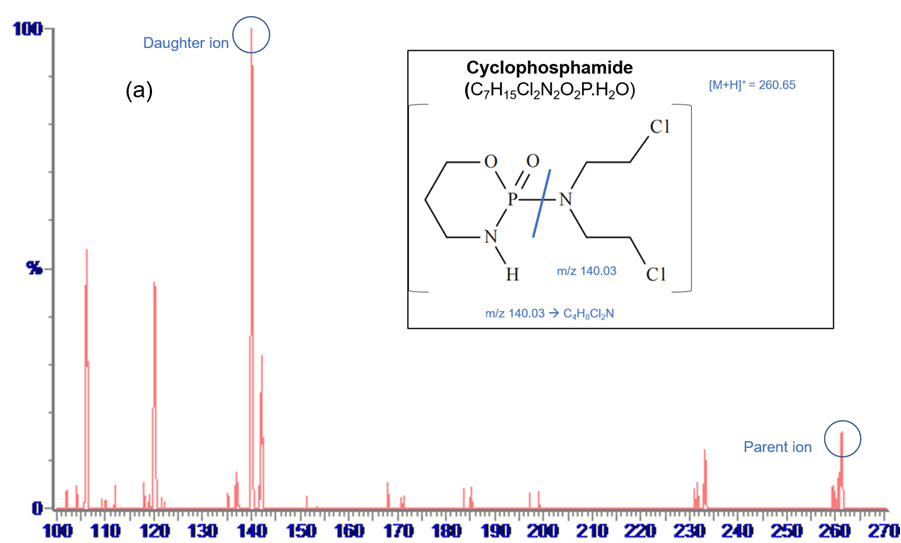
**

**
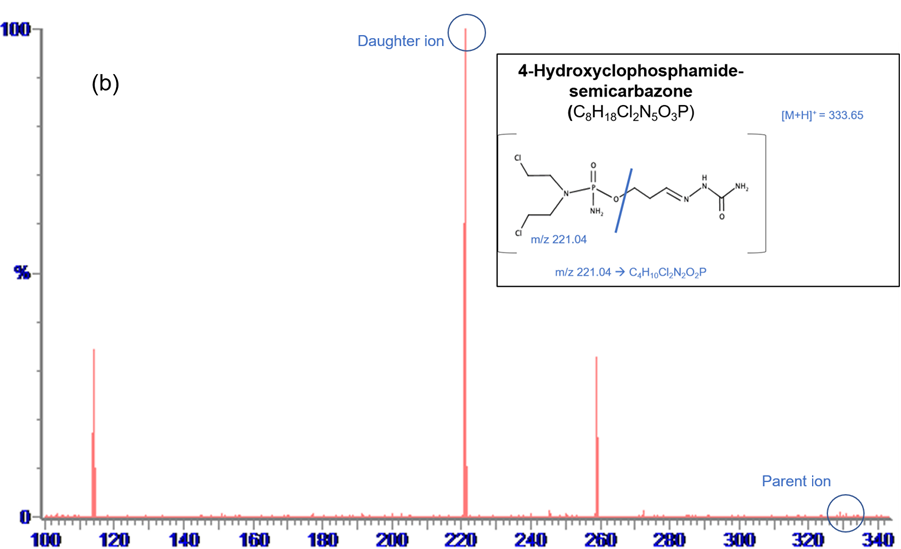
**

**
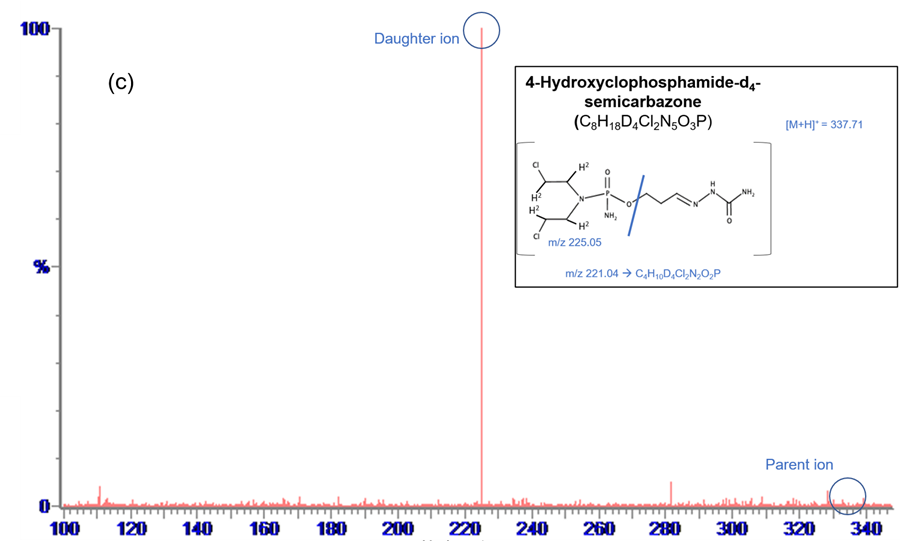
**

**Figure S1** Fragmentation spectrum of (a) cyclophosphamide, (b) 4-hydroxycyclophosphamide-semicarbazone, and (c) 4-hydroxycyclophosphamide-d_4_-semicarbazone.

**
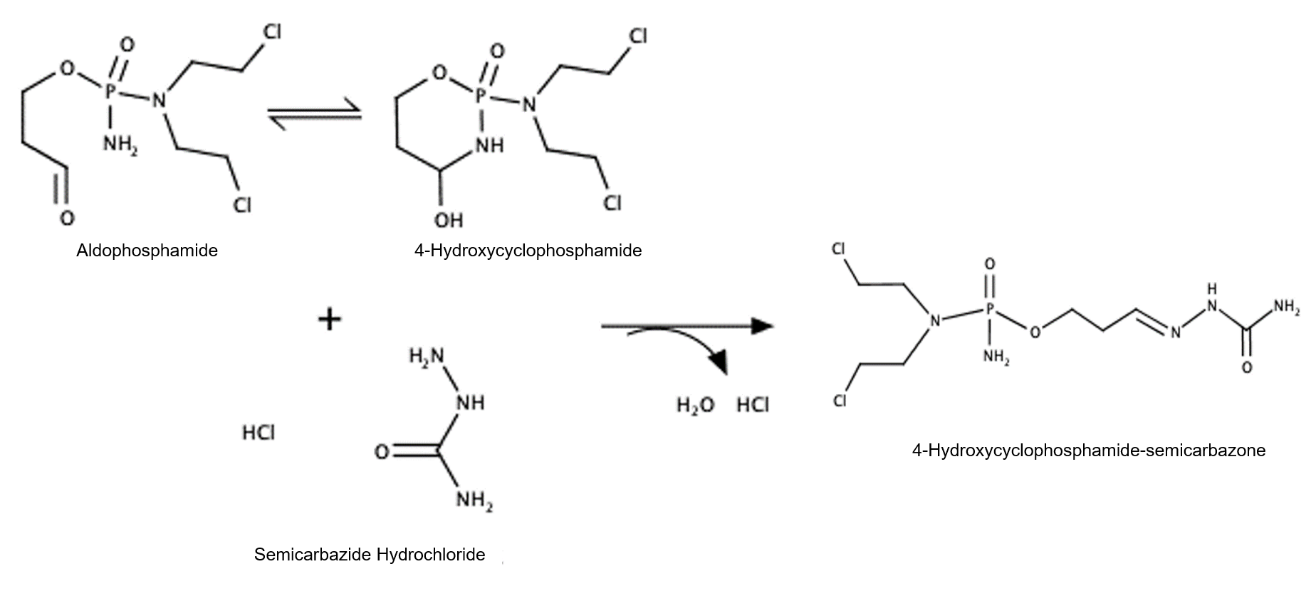
**

**Figure S2** The derivatization reaction of aldophosphamide/4-hydroxycyclophosphamide with semicarbazide hydrochloride**.**
